# Hospice Family Caregivers’ Use of Audio Diaries

**DOI:** 10.1093/geroni/igaa057.785

**Published:** 2020-12-16

**Authors:** Kristin Cloyes, Eliabeth Porter, Maija Reblin, Kathi Mooney, Djin Tay, Lee Ellington

**Affiliations:** 1 University of Utah, Portland, Oregon, United States; 2 University of Utah, Salt Lake City, Utah, United States; 3 Moffitt Cancer Center, Tampa, Florida, United States

## Abstract

Previous work has shown that emotional processing as part of diary writing improves well-being during and after stress. The purpose of this study was to determine the feasibility of verbal/audio diaries for home hospice family caregivers (HFCGs). We also describe diary content. As part of an ongoing multi-site, prospective longitudinal study, HFCGs of cancer patients report daily fluctuations in patients’ and their own symptoms via an automated telephone system, including a recorded diary entry. HFCGs are randomly assigned instructions to either discuss additional symptoms or discuss their thoughts and feelings. Thirty-six (85.7%) participants to date have completed at least one audio diary. For this preliminary analysis, we selected the 14 longest diary recordings from each condition (n=28) to content analyze using Linguistic Inquiry and Word Count (LIWC) and NVivo 12. Participants are 78.6% female, 53 years of age on average, and most are spouse/partner (46.4%) or adult child (35.7%) caregivers. There was no difference in the overall positivity (23%) or negativity (77%) of words in either condition, but participants asked to express thoughts/feelings used significantly more anger-related terms (p=0.04) while those asked to describe symptoms used significantly more anxiety-related terms (p = 0.003). Time was the most common theme in both conditions but arose more frequently in the symptom condition (p=.08). Our findings suggest that most audio diaries are feasible for HFCGs, but varied prompts may facilitate different types of emotional expression. Future research should assess potential impact on emotional well-being and bereavement adjustment.

